# Edema of the Ligamentum Teres as a Novel MRI Marker for Non-Traumatic Painful Hip Pathology: A Retrospective Observational Study

**DOI:** 10.7759/cureus.23388

**Published:** 2022-03-22

**Authors:** Syed Alam, Amman Yousaf, Yahya Alborno, Mohammad Shujauddin, Syed Imran Ghouri, Basel Abdelazeem, Ahmad L F Yasin, Syeda Shabistan, Ghalib Ahmed

**Affiliations:** 1 Musculoskeletal Radiology, Hamad Medical Corporation, Doha, QAT; 2 Internal Medicine, McLaren Flint, Flint, USA; 3 Orthopaedics, Hamad Medical Corporation, Doha, QAT; 4 Radiology, Hamad Medical Corporation, Doha, QAT; 5 Department of Radiology, Jawaharlal Institute of Postgraduate Medical Education and Research, Pondicherry, IND

**Keywords:** hip joint, teres, ligamentum, role of mri, femoral acetabular impingement, instability

## Abstract

Background

The ligamentum teres has been recognized as an important stabilizer of the hip joint and can be affected by various hip pathologies. This study aims to introduce ligamentum teres edema as an MRI marker to diagnose the underlying cause of hip pathology, mainly femoral acetabular impingement (FAI) and adult developmental dysplasia of the hip (ADDH), in non-traumatic patients.

Methodology

Adult patients presenting with non-traumatic hip pain of variable duration and ligamentum teres edema on MRI between 2014 and 2020 were included. A high-resolution standard MRI hip protocol was used for all patients in this series. MRI and plain radiographs were assessed. Ligamentum teres edema, alpha angle, center edge angle of Wiberg, and retroversion were assessed.

Results

In total, 55 patients with 110 hip joints (males: 29 (52.7%), females: 26 (47.3%)) of different ethnicities were included in this study. Out of the 55 patients with ligamentum teres edema, one had only unilateral right-sided FAI, seven had only unilateral left-sided FAI, and 46 (94 hip joints) had either bilateral FAI or ADDH. Therefore, eight (14.5%) patients with unilateral FAI had the absence of the contralateral FAI or ADDH (6.5% false-positive) despite the presence of ligamentum teres edema bilaterally, and the rest of the patients with bilateral ligamentum teres edema (102 joints: 92.7% positive predictive value) had findings of either FAI or ADDH.

Conclusions

Ligamentum teres edema can be considered as an early MRI marker to diagnose the underlying pathology of symptomatic painful hip disorders, especially FAI.

## Introduction

Ligamentum teres is an intra-articular extra-synovial structure consisting of two to six distinct bundles originating along the length of transverse acetabular ligament and inferior aspect of the cotyloid fossa [1]. Its structure and function have been studied profoundly in recent literature. It has been described as a secondary stabilizer of the hip that can prevent painful and excessive hip movements [2]. Due to its tensile strength, it contributes to the prevention of hip dislocation and micro-instability, and its role has been acknowledged as being comparable to the anterior cruciate ligament [2,3]. In recent literature, ligamentum teres tear, which is usually diagnosed by hip arthroscopy, was considered as one source of hip pain. Various associations have been hypothesized between ligamentum teres pathologies and other hip pathologies [2,4].

The purpose of this study is to introduce ligamentum teres edema as an MRI marker to possibly diagnose the underlying cause of hip pathology, mainly femoral acetabular impingement (FAI) and adult developmental dysplasia of the hip (ADDH)c, in non-traumatic patients.

## Materials and methods

Patients

In this study, single-center institutional data for six years (from 2014 to 2020) was used. Institutional Review Board approval was waived owing to the anonymized use of patients’ data.

Inclusion and exclusion criteria

Adult patients presenting with non-traumatic hip pain of variable duration who underwent MRI of the hip and had ligamentum teres edema were included. Patients presenting with traumatic hip pain, previous hip surgery, and the lack of clear MRI images were excluded from the study.

MRI technique and interpretation

A high-resolution standard MRI hip protocol was used for all patients in this series. MRI was performed using a 3-T and 1.5-T Siemens MRI machine. MRI scans of these patients were reviewed retrospectively by a senior musculoskeletal radiologist. Axial and coronal T1-weighted images (T1WI) without fat saturation, axial T2WI with fat saturation, sagittal T2WI with fat saturation, short tau inversion recovery (STIR) coronal images, and proton density (PD) fat saturation oblique axial images were reviewed. Ligamentum teres edema was assessed on oblique axial PD fat saturation and STIR coronal sequences. Ligamentum teres edema was considered significant when there was an intermediate/high signal intensity with thickening of the ligamentum teres. Alpha angle (on oblique axial), center edge angle of Wiberg (coronal T1WI without fat saturation or anteroposterior (AP) pelvis radiograph), and retroversion (on axial T1WI or T2WI or plain X-ray AP view) were assessed. Alpha angle was considered abnormal when it was above 60 degrees, center edge angle of Wiberg was considered abnormal when it was below 25 or above 40 degrees. This article has been reported in line with Strengthening the Reporting of Observational Studies in Epidemiology (STROBE) guidelines [5].

## Results

In total, 55 patients with 110 hip joints (males: 29 (52.7%), females: 26 (47.3%)) of different ethnicities were included in this study. Out of the 55 patients, 19 (34.5%) presented with chronic left hip pain, 19 (34.5%) with chronic right hip pain, and 17 (30.9%) with chronic bilateral hip pain. A radiological assessment was performed for the 110 hip joints. Out of the 55 patients with ligamentum teres edema, one had only unilateral right-sided FAI, seven had unilateral left-sided FAI, and 47 (94 hip joints) had either bilateral FAI or ADDH. FAI presented in 47 (92%) cases while ADDH presented in 7.8% of the cases. Eight (14.5%) patients with unilateral FAI had a contralateral absence of FAI/ADDH (7.28% false-positive) despite the presence of ligamentum teres edema bilaterally, and the rest of the patients (103 joints: 92.7% positive predictive value) had findings of either FAI or ADDH (Table 1).

**Table 1:** PPV of ligamentum teres edema in FAI/ADDH diagnosis. FAI: femoral acetabular impingement; ADDH: adult developmental dysplasia of hip; PPV: positive predictive value

	FAI/ADDH positive	FAI/ADDH negative	PPV (%)
Ligamentum teres edema positive	103	7	92.72

Out of the 92.72% patients with bilateral ligamentum teres edema and FAI/ADDH, 56.86% (58/102) had cam-type FAI, 8.82% (9/102) had pincer FAI, 26.47% (27/102) had mixed FAI, and 7.84% (8/102) had ADDH (Table 2). The left side (68.5%) was more severely affected than the right (31.5%).

**Table 2:** Demographics and clinical presentation of ligamentum teres edema in patients with non-traumatic adult hip pain. MRI: magnetic resonance imaging; FAI: femoral acetabular impingement; ADDH: adult developmental dysplasia of the hip

Age (mean ± SD)	41.7 ± 9.9
Total number of patients	n = 55
Male	29 (52.7%)
Female	26 (47.3%)
Side of hip pain	
Right	19 (34.5%)
Left	19 (34.5%)
Bilateral	17 (30.9%)
Average duration of symptoms	Six months
MRI features	
Number of hip joints reviewed	110
Ligamentum teres edema	110
Sides involved (hip joints)	
Right-sided FAI	1
Left-sided FAI	7
Bilateral FAI/ADDH	94
Types of abnormalities	
CAM FAI	58 (56.86%)
Pincer FAI	9 (8.82%)
Mixed FAI	27 (26.47%)
ADDH	8 (7.8%)

## Discussion

In this study, 92.7% of non-traumatic chronic hip pain patients had ligamentum teres edema in both hip joints on MRI images bilaterally. However, 6.5% of the patients had ligamentum teres edema in the joint without having an internal hip pathology or even pain in the affected hip. Overall, 92% of patients in this study had FAI. The prevalence of ligamentum teres tears in the asymptomatic cohort has been reported to be as low as 2.2% based on MRI evaluation [6]. Ligamentum teres abnormalities are considered one of the most common causes of hip pain in adults and are usually associated with other underlying hip pathologies [7]. Recent studies have shown an association between ligamentum teres tears on MRI and bony acetabular morphology [8]. Park et al. reviewed MRI images in a patient cohort and found that insufficient acetabular coverage is associated with a complete tear of the ligamentum teres [8]. Further, the presence of ligamentum teres tears is associated with distinct articular cartilage damage [1].

Ligamentum teres edema was observed in all patients in this study using non-contrast MRI of the hip. The normal appearance of the ligamentum teres on MRI consists of discrete-continuous bundles with smooth edges, normal insertion, and homogenous hypointense signal on T1WI and T2WI [1]. Hyperintense T2 signal with ligament irregularity and partial discontinuity of the fibers was detected with a partial tear [1,8]. Recent MRI advancements have improved the accuracy of diagnosing different ligamentous injuries. Devitt et al. found an overall accuracy of 64% in the diagnosis of partial tears using a 3-T MRI with no arthrography (sensitivity, 9%; specificity, 91%) [9]. A systematic review reported that the sensitivity and specificity of all MRI examinations were 64.7% and 86.9%, respectively [10]. Early-stage FAI or ADDH was diagnosed in patients with ligamentum teres edema with a PPV of 92.7% which is quite significant. Ligamentum teres tears are prevalent in developmental dysplasia of the hip and the presence of cam impingement [1]. At the time of the diagnosis of FAI or ADDH, all patients in this study were symptomatic with chronic unilateral or bilateral hip pain for an average of six months.

**Figure 1:**
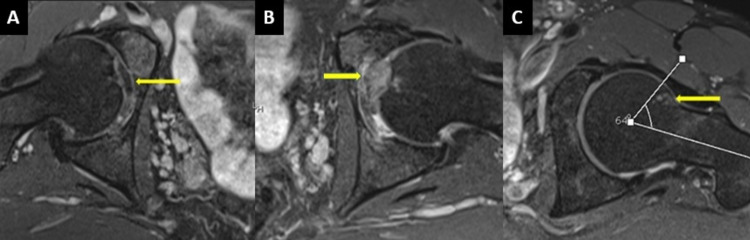
MRI (oblique axial PD fat saturation) image of a 47-year-old male who presented with chronic left-sided hip pain. (A) Normal ligamentum teres on the right side (yellow arrow). (B) Left ligamentum teres edema and hypertrophy. (C) The alpha angle is increased by 64 degrees, and fibrocystic changes are seen in the head-neck junction with a small bony bump in the left hip. MRI: magnetic resonance imaging; PD: proton density

The majority of patients in this study were diagnosed with FAI (92%) with a higher prevalence of cam-type lesions. FAI is an important cause of hip pain and impingement in young adults that, if not addressed, might lead to a painful osteoarthritic joint [4,11]. Plain radiographs and MRI scans can be used to diagnose FAI. Alpha angle was first described on MRI to demonstrate the degree of sphericity of the femoral head in cam-type FAI [12]. Later, this measure was applied to plain radiographs and computed tomography. An alpha angle above 60 degrees in symptomatic patients is considered diagnostic for cam-type FAI [11,13] (Figure 1).

**Figure 2:**
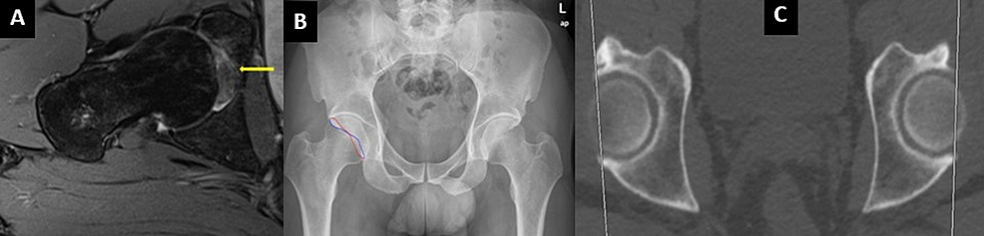
A 45-year-old male who presented with right-sided hip pain. (A) MRI oblique axial PD fat saturation image showing ligamentum teres edema and thickening. (B) X-ray AP pelvis demonstrates bilateral retroversion, more severe on the right side. It shows the figure of 8 appearance (blue: posterior acetabular rim, red: anterior acetabular rim). (C) Axial CT of the hip depicting bilateral retroversion, more severe on the right side. MRI: magnetic resonance imaging; PD: proton density; CT: computed tomography

On the AP pelvis view, the acetabular retroversion (the anterior acetabular rim crossing over the posterior rim) should be ruled out (Figure 2).

**Figure 3:**
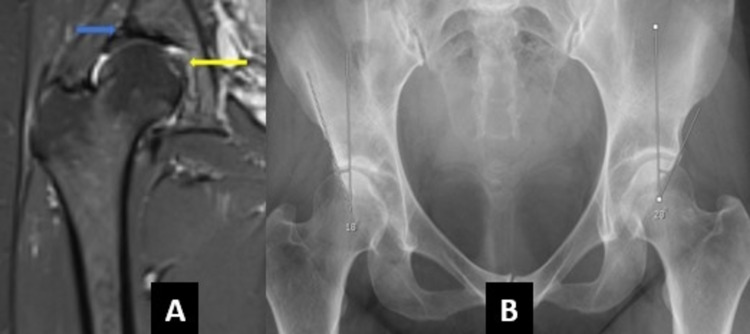
A 46-year-old patient who presented with right hip pain. (A) Coronal STIR sequence of MRI demonstrating ligamentum teres edema (yellow arrow) with the hypertrophy of the labrum (blue arrow). (B) AP pelvis view showing decreased center edge angle (18 degrees) on the right side. STIR: short tau inversion recovery; MRI: magnetic resonance imaging

ADDH was observed in a small number of patients in this study which can be diagnosed by reviewing radiographs. weight-bearing AP, and false profile views of the pelvis. The center-edge angle of more than 25 degrees and Tonnis angle below 10 degrees rule out hip dysplasia [12,14,15] (Figure 3).

With technological advancements and the availability of 3-T MRIs, ligamentum teres edema can be easily observed in subtle cases without contrast or arthrogram. Hip arthroscopy remains a better tool for diagnosing ligamentum teres abnormalities; however, MRI is a non-invasive tool to detect a wide range of hip pathologies [16,17].

Limitations

Because this was a retrospective study, there may be some bias. Moreover, the lack of an asymptomatic control group with hip MRI might affect the accuracy of the study findings.

## Conclusions

Ligamentum teres has been recognized as an important stabilizer of the hip joint, and changes in the ligamentum teres can be a crucial clue to highlighting other hip abnormalities. We propose that ligamentum teres edema can be an important early MRI marker to diagnose the underlying pathology of symptomatic painful hip disorders, especially FAI. Moreover, the majority of patients with ligamentum teres edema and FAI were symptomatic in our study; therefore, we suggest that though FAI can be asymptomatic, patients presenting with ligamentum teres edema will be almost always symptomatic. This study warrants further controlled studies in detecting the accuracy of this marker in detecting hip abnormalities.
